# A Prototype Intraoral Periapical Sensor with High Frame Rates for a 2.5D Periapical Radiography System

**DOI:** 10.1155/2019/7987496

**Published:** 2019-04-24

**Authors:** Che-Wei Liao, Ker-Jer Huang, Jyh-Cheng Chen, Chih-Wei Kuo, Yin-Yi Wu, Jui-Ting Hsu

**Affiliations:** ^1^Graduate Institute of Biomedical Sciences, China Medical University, Taichung 404, Taiwan; ^2^Materials & Electro-Optics Research Division, National Chung-Shan Institute of Science & Technology, Taoyuan City 407, Taiwan; ^3^Department of Biomedical Imaging and Radiological Sciences, National Yang-Ming University, Taipei 112, Taiwan; ^4^School of Dentistry, College of Dentistry, China Medical University, Taichung 404, Taiwan; ^5^Department of Bioinformatics and Medical Engineering, Asia University, Taichung 413, Taiwan

## Abstract

X-ray radiography is currently used in dentistry and can be divided into two categories: two-dimensional (2D) radiographic images (e.g., using periapical film, cephalometric film, and panoramic X-ray) and three-dimensional (3D) radiographic images (e.g., using dental cone-beam computed tomography (CBCT)). Among them, 2D periapical film images are most commonly used. However, 2D periapical film compresses 3D image information into a 2D image, which means that depth cannot be identified from the image. Such compressed images lose a considerable amount of information, reducing their clinical applicability. A 2.5D periapical radiography system prototype was developed by our research team. Our previous study indicated that this prototype could be used to capture images at different depths of an object. However, the prototype was limited by its commercially available intraoral periapical sensor, which had a low temporal resolution and could not capture multiple images in a short period of time. Therefore, the total time required for image capture was too long for practical clinical application. The present study developed a high-frame-rate intraoral periapical sensor with a sensor imaging speed of up to 15 Hz. The primary components of the developed intraoral periapical sensor include a scintillator, complementary metal oxide semiconductor chip, component circuit board, and video processing board. The external dimensions of the sensor are 41 × 26 × 6.6 mm^3^. The performance of the developed high-frame-rate intraoral periapical sensor was verified through qualified and quantified analyses using line pairs. The results showed that the resolution of the developed intraoral periapical sensor could reach 18 lp/mm. The sensor was further installed in our 2.5D periapical radiography system to conduct image capturing. The results indicated that the developed sensor could be used for high-frame-rate imaging to incorporate tomosynthesis to obtain reconstructed slice images of different depths. The developed sensor has the potential for clinical dentistry applications in the future.

## 1. Introduction

Since X-ray was discovered by Wilhelm Conrad Röntgen more than 100 years ago, it has been widely applied in medicine. X-ray can be used for noninvasive medical examinations and is one of the methods often used to assess hard tissue before surgery [1]. X-ray imaging requires a sensor to capture the image. In the early days of X-ray technology, radiographic films were applied to capture images. In the past 30 years, sensors for X-ray imaging have gradually been digitalized [2], and digital radiography can now be divided into three categories, namely, computed radiography (CT), indirect digital radiography, and direct digital radiography [3, 4]. In dentistry, the most common application of X-rays is periapical film, which has advantages such as high resolution, easy operation, and low costs [5, 6]. However, periapical film can only provide two-dimensional (2D) images; specifically, three-dimensional (3D) tissue compressed projection can only be displayed in a 2D image. In such 2D images, 3D tissues with different depths are relatively difficult to distinguish, and image distortion may be caused in the compression process, thereby often limiting the clinical applicability [7, 8].

To overcome the shortcomings of image compression in periapical film, scholars have conducted experiments using tomosynthesis in imaging [9]. In 2013, Li et al. [7] conducted a laboratory experiment with a pig's mandible. A film was placed on the posterior mandible, and the X-ray tube was rotated at a limited angle of ±30°. These 2D projection images were reconstructed through tomosynthesis to obtain numerous slice images of different depths. Shan et al. [10] and Inscoe et al. [11, 12] used a carbon nanotube X-ray source array to develop stationary digital tomosynthesis for dental imaging. In their studies [10–12], the feasibility of stationary intraoral tomosynthesis was demonstrated. They built prototype stationary intraoral tomosynthesis imaging systems, which were evaluated and found to meet all the manufacturers' specifications. In addition, our team built a prototype of a 2.5D periapical radiography system in 2018 [13]. Adopting the tomosynthesis approach, we placed a canine in front of a commercially available intraoral periapical sensor and rotated the X-ray tube (±60°) to obtain various 2D projection images. The images were reconstructed using tomosynthesis. Our results proved that tomosynthesis can be applied in dentistry to obtain slice images of different depths [14–16]. However, in the experiment, we also found that the commercially available intraoral periapical sensor possessed relatively low temporal resolution and that the system could not continuously capture images multiple times in a short period. Because the original purpose of the commercially available intraoral periapical sensor was not continuous capture of high-frame-rate images, it took tens of minutes to capture a set of images, making it unsuitable for clinical application. Therefore, in order for our proposed 2.5D periapical radiography system to be used as a diagnostic tool in dentistry, it requires a high temporal resolution sensor that can continuously capture multiple images in a short period.

Currently, high-frame-rate sensors are used in technology such as CT, CBCT, and micro-CT [5, 17, 18], all of which employ large sensors that cannot be placed in the mouth. Therefore, this study developed a high-frame-rate intraoral periapical sensor capable of frame rates of up to 15 Hz. The developed sensor was applied to the 2.5D periapical radiography system developed by this research team.

## 2. Materials and Methods

**Figure 1:**
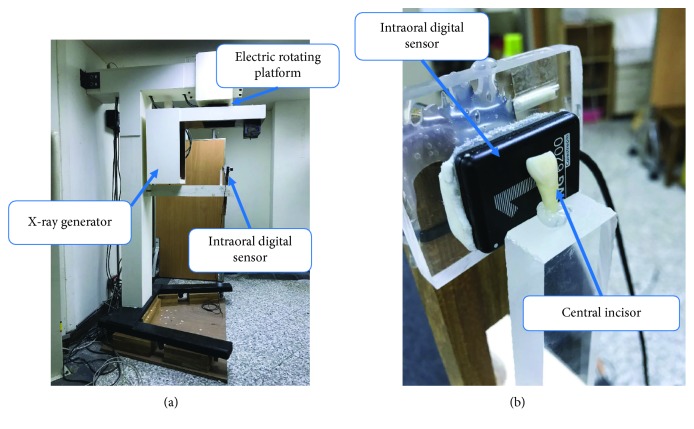
Prototype of the intraoral digital tomosynthesis system: (a) entire view and (b) close view (figure reproduced with permission).

In our previous study [13], we developed a prototype 2.5D periapical radiography system (Figure 1) combining a commercially available intraoral periapical sensor, an X-ray tube, and a supporting frame. A total of 121 canine 2D projection images were taken at a limited angle of ±60°. The images were reconstructed using tomosynthesis to obtain slice images of the canine at different depths. However, the commercial intraoral periapical sensor used in the prototype requires cooling for a few seconds between takes to avoid overheating. The process of capturing a set of images in the range of ±60° therefore took tens of minutes to complete, making the system unsuitable for clinical applications. The present study therefore designed a high-frame-rate intraoral periapical sensor capable of frame rates of up to 15 Hz. The proposed sensor could greatly shorten the shooting interval, thereby enhancing its clinical applicability.

### 2.1. Components of the High-Frame-Rate Intraoral Periapical Sensor

**Figure 2:**
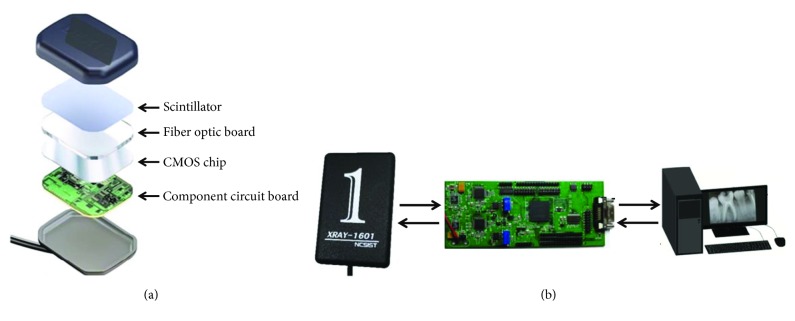
(a) The major components of the high-frame-rate intraoral periapical sensor and (b) the high-frame-rate intraoral periapical sensor, video processing board, and control computer.

The intraoral component of the proposed high-frame-rate intraoral periapical sensor can be divided into four parts, namely, the scintillator, complementary metal oxide semiconductor (CMOS) chip, component circuit board, and video processing board (Figure 2). The top layer is the scintillator. The scintillator converts the X-ray intensity to visible light of different grayscales, and the visible light signals are then converted from photon signals to electric signals through the coupling layer and complementary metal oxide semiconductor (CMOS) sensor array. Subsequently, the electric signals from the CMOS sensor array are transmitted to a video processing board (outside the subject's mouth) through a USB 3.0 cable. The electric (analog) signals of the CMOS sensor array are then converted into digital signals, which are transmitted to the computer for imaging (Figure 2).

#### 2.1.1. Process of Scintillator

CsI scintillator columns exhibit high absorption capacity for X-rays. These columns can be applied in scintillation detectors for capturing simultaneous short-wave images as well as for digitalizing images. A pure CsI scintillator has an extremely short luminescence decay time (3.5 ns); however, incorporation of a thallium activator can greatly improve the crystal luminescence efficiency, thereby facilitating coupling between the optical emission and photomultiplier. In addition to enhancing the photoelectron conversion efficiency of the scintillator, changing the refractive index of the X-ray in the anodic aluminum oxide template can increase the residence time of the X-ray in the CsI(Tl) crystal and improve the photoelectron conversion efficiency of the scintillator.

**Figure 3:**
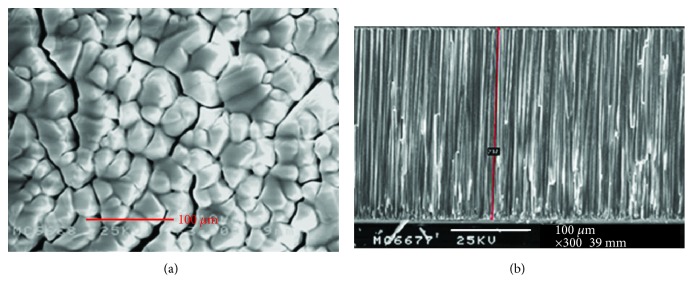
Scanning electron microscope images of the thin-film scintillator: (a) top view and (b) cross-sectional side view.

In this study, we used the thermal evaporation method and adjusted the controlling process parameters to grow a columnar CsI film. After the CsI(Tl) powder was uniformly mixed, the powder was made into a compressed tablet by using a tablet press machine and sintered to remove moisture and impurities in the powder through annealing. The powder can be made more compact through the compressing process, thereby reducing the amount of air in the tablet. The purpose of the tablet compression was to prevent the air from being rapidly expanded due to the heat during the evaporation process, which may have caused powder splash that would affect the quality of the scintillator. Annealing using different temperature parameters can effectively eliminate thin-film cracking, formation of voids on the film, and a disordered structure of the interface (Figure 3).

#### 2.1.2. CMOS Chip and Component Circuit Board

**Figure 4:**
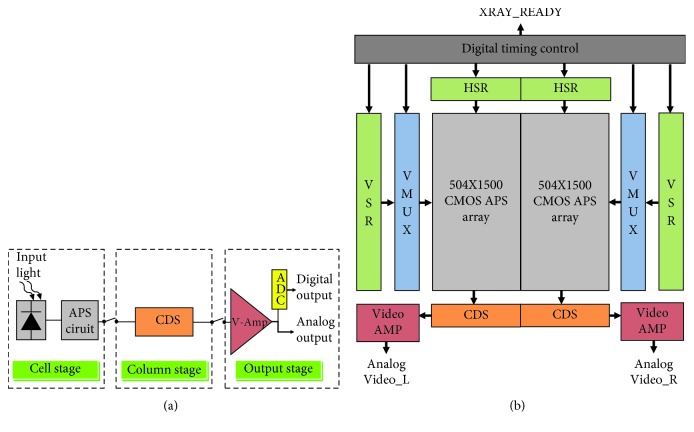
(a) CMOS image sensor signal processing flow chart and (b) architecture of the chip export signals.

Once the scintillator produces visible light, the optical signals are transmitted to the CMOS image sensor by the continuous shooting fiber optic board. The CMOS is a mixed integrated circuit that includes an analog circuit and a digital circuit. The analog circuit processes optical signals and includes four electric circuit modes, namely, a photon-to-electron transformation circuit (active pixel sensor (APS)), pixel signal collection circuit (correlated double sampling (CDS)), pixel signal amplification circuit (video output amplifier (V-Amp)), and analog-to-digital conversion circuit (ADC). CMOS image sensing utilizes the photoelectric effect to excite electrons in the silicon crystal from the valence to conduction bands, and the optical signal strength is measured by the amount of photocurrent generated during the process. The CMOS image sensor uses the N+-to-PSUB PN interface as a light sensor.  Figure 4(a) shows a flow chart of the signal processing of the CMOS image sensor. The architecture of the CMOS chip signal export is illustrated in Figure 4(b). To improve the image export rate, the chip signal is synchronously processed by a dual-channel setting and exported through Analog Video_L and Analog Video_R. Regarding the circuit of the AD9244 analog-to-digital converter in the video processing board, V_Out1 is the chip output signal of one of the channels; however, it is also the input signal for the video processing board. After being transmitted through the sample-and-hold circuit, the signal is transmitted to the AD9244BSTZ-40 chip, and the final output digital image data format is 14 bits.

#### 2.1.3. Video Processing Board

The chip imaging architecture requires a matching video processing board. The primary function of the matched video processing board is to output signals to the A/D converter chip on the external video processing board through the serial transmission of chipsets. Subsequently, the video processing board converts the signals into digital signals and transmits them through CameraLink, a high-speed data transmission interface, to output the image information to a computer for data storage. The image output format is TIFF, the image size is 1000 × 1496 pixels with a pixel size of 20 *μ*m, and the image format is 14 bits. Another function of the video processing board is to synchronously control the X-ray tube. Because the video processing board controls the X-ray tube exposure, the board also controls the synchronization timing of the chipset exposure, meaning that the chipset and X-ray tube can be synchronously activated to accurately control the chipset and begin receiving signals.  Table 1 lists the characteristic features of the developed high-frame-rate intraoral periapical sensor.

### 2.2. Quantitative Performance of the High-Frame-Rate Intraoral Periapical Sensor

The developed intraoral periapical sensor was also used to capture images of two phantoms to verify image quality. The phantoms were line pairs. From the images of the line pairs, the calculated modulation transfer function (MTF) could be used to measure the actual resolution of the sensor.

### 2.3. Applying the High-Frame-Rate Intraoral Periapical Sensor in the Prototype 2.5D Periapical Radiography System

**Figure 5:**
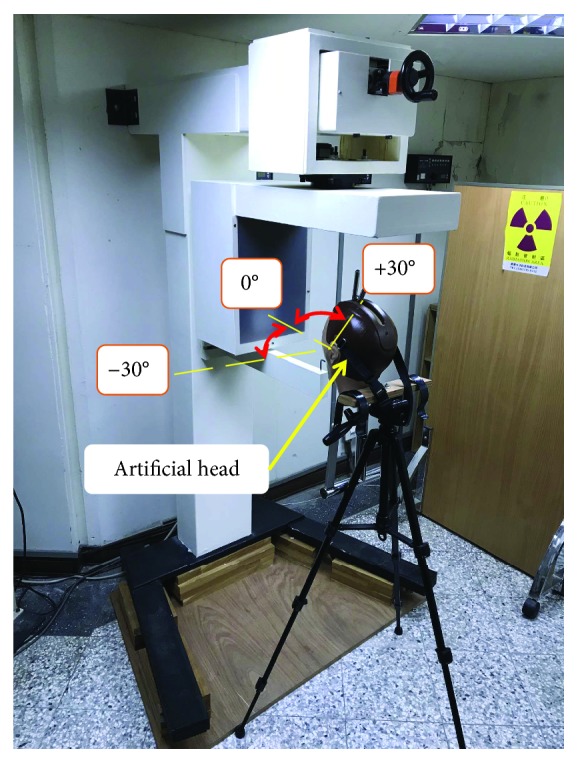
2.5D periapical radiography system and X-ray tube scanning ranges.

We installed the high-frame-rate intraoral periapical sensor on our previously developed 2.5D periapical radiography system prototype (Figure 5) [13]. The image sample was a human third molar. For details regarding the prototype, refer to our previous study [13]. The scanning parameters were as follows: distance between the X-ray source and rotation axis was 350 mm; distance between the sensor and rotation axis was 5 mm; the voltage was 80 kVp; the current was 5 mA; exposure time was 0.2 second; the angle of the X-ray tube was ±30°; and images were taken every 2°. A total of 31 images were taken, and these 31 2D projection images were used to reconstruct images through tomosynthesis.

## 3. Results

### 3.1. The Performance of the High-Frame-Rate Intraoral Periapical Sensor

**Figure 6:**
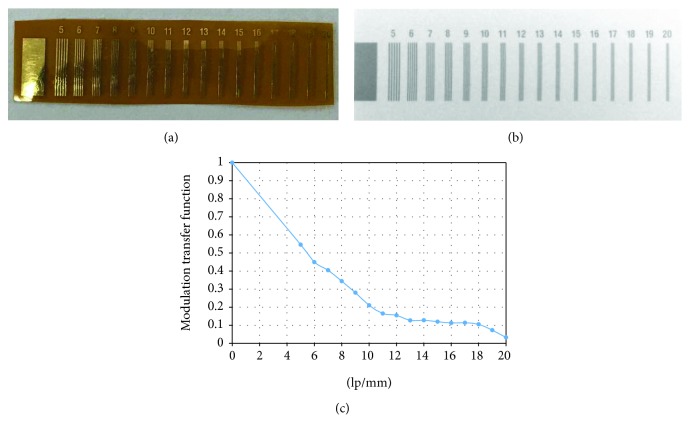
(a) The line pair phantom, (b) the image of the line pair phantom, and (c) the curve of modulation transfer function.

This high-frame-rate intraoral periapical sensor utilized two methods for image quality evaluation, namely, line pairs and an aluminum step wedge. From the line pair images (Figure 6(a)), image quality was quantized using the MTF, the value of which was lower than 0.09 at 19 lp/mm (Figure 6(b)); thus, the resolution of the developed intraoral periapical sensor was 18 lp/mm.

### 3.2. The Performance of the 2.5D Periapical Radiography System Using the High-Frame-Rate Intraoral Periapical Sensor

**Figure 7:**
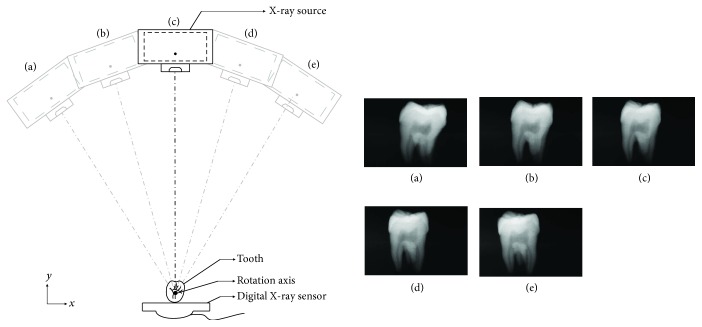
X-ray tube captures 2D projection images of the third molar at different angles.

The high-frame-rate intraoral periapical sensor was installed in the 2.5D intraoral periapical sensor prototype designed by our research team. The X-ray tube scanned the 2D projection images of the human third molar at an angle of 30°.  Figure 7 illustrates that the more the X-ray tube deviates from the orthogonal axis (0°) of the sensor, the more severe the deformation of the 2D projection images becomes. At ±30°, an image was captured every 2°, for a total of 31 images taken in approximately 4 s. Clear outlines of the dentin and enamel of the third molar can be observed in each of the 2D projection images; thus, the outline was not distorted or blurred when shooting at a high frame rate.

**Figure 8:**
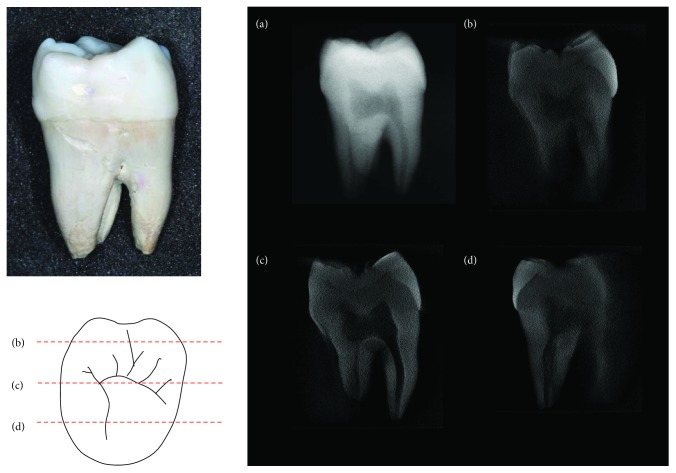
(a) Periapical radiology image of the third molar and (b–d) the reconstructed slice images at different depths from the sensor surface.

The 2D projection images captured by the X-ray tube at 0° were equivalent to the images captured using clinical periapical film. In images captured using periapical film, 3D images of tissue are compressed into a 2D image (Figure 8(a)). Figures 8(b)–8(d) present the reconstructed images utilizing the 31 2D projection images taken of the third molar at different depths. These images display the anteroposterior relationship between different parts of the molar, and the structure of the internal tissue, such as the dentin, enamel, and pulp cavity, is also present in the images. These images provide more information regarding the molar than the 2D periapical film image does (Figure 8(a)).

## 4. Discussion

Our research team previously conducted in vitro tests to verify the feasibility of our 2.5D periapical radiography system [13]. By using an X-ray tube, an intraoral periapical sensor, a supporting frame, and electronic control equipment, we captured multiple 2D projection images of a tooth. These projection images were used to reconstruct images by adopting tomosynthesis to obtain slice images of different depths of the tooth. Images captured using this method provided more image information than those captured with conventional periapical film, and the images from the 2.5D periapical radiography system were not affected by the image superposition that occurs with a 2D periapical film. However, we also found that the 2.5D periapical radiography system prototype was unsuitable for current clinical applications because the temporal resolution of the commercially available intraoral periapical sensor was low and the sensor required a few seconds between each shot, resulting in an overall shooting time of tens of minutes. Therefore, the present study developed a high-frame-rate intraoral periapical sensor with a frame rate of up to 15 Hz. The preliminary test results indicated that the system could perform a tomosynthesis scan of ±30° in only 4 s, which greatly improves the potential for clinical application of our 2.5D periapical radiography system.

Periapical film has the advantages of high resolution, easy operation, and low costs [6]; however, its ultimate limitation is that it can only capture 2D images. Our research team previously showed the feasibility of a 2.5D periapical radiography system [13] using a commercial sensor (RVG6200-SIZE1, Carestream Dental, Stuttgart, Germany) and an X-ray tube of ±60° to obtain 2D projection images every 1°. The obtained 2D projection images of a canine were then used to reconstruct images of the canine at different depths. The reconstructed images obtained using tomosynthesis were similar to those obtained by using an X-ray tube to perform 360° scanning incorporating the background projection method; the dentin and enamel outlines could be distinguished in the images. In a previous study [13], we demonstrated that tomosynthesis could be applied to dentistry. As early as 1996, Webber et al. [19] had already used an intraoral CCD X-ray transducer to indicate that tomosynthesis might be applicable to dentistry. In 2013, Li et al. [7] constructed a desktop intraoral digital tomosynthesis system in the laboratory and conducted an experiment on a pig mandible. By placing a sensor in the posterior mandible, the results of their study also indicated the feasibility of tomosynthesis in dentistry. Currently, tomosynthesis is mostly applied to mammography [15] [20], with no commercially available products found in dentistry. A possible reason for this is that no high-frame-rate intraoral periapical sensor currently exists. Therefore, this study sought to develop such a sensor.

Currently, high-frame-rate sensors with imaging speeds of up to 10 Hz are available [21, 22]; however, these are mostly used in CT, dental CBCT, or micro-CT [23–25]. Moreover, these sensors are at least 12 × 7 or 15 × 15 cm^2^, which means they are too large to be placed in the mouth. Intraoral periapical sensors used in modern dentistry are not designed for high-frame-rate capture; thus, manufacturers do not provide information on maximum sensor frame rates. Our previous study found that interval between shots of less than 5 s using a commercial sensor led the system to overheat. Therefore, development of a high-frame-rate intraoral periapical sensor is necessary if our 2.5D periapical radiography system is to be used for future clinical applications. In addition to use in our 2.5D periapical radiography system, the high-frame-rate intraoral periapical sensor developed in the present study can be applied to a micro-CT machine [13] or used in industrial quality management that requires capturing high-frame-rate images.

The line pair phantom was used in this study to quantify the image quality. According to the analysis results, the resolution of the intraoral periapical sensor was 18 lp/mm. In our previous study [13], we employed the RVG6200-SIZE1 commercial intraoral digital sensor (Carestream Dental, Stuttgart, Germany) to build a prototype 2.5D periapical radiography system. The line pair phantoms were used to measure the resolution of the commercial sensor, with a result of 18 lp/mm, which was similar with the developed high-frame-rate intraoral periapical sensor.  Figure 7 displays 2D projection images of the third molar captured by the X-ray tube at different angles. Because the high-frame-rate intraoral periapical sensor was fixed in place, the larger the shooting angle of the X-ray tube was, the more severe the image distortion of the teeth became. However, the outlines and boundaries of the dentin and enamel on these distorted images could still be identified. These projection images were taken by the moving X-ray tube, and due to the high-frame-rate capture, each image was blur free.

In this study, the images reconstructed from the 31 2D projection images using tomosynthesis revealed slice images of the third molar at different depths (Figures 8(b)–8(d)). Compared with 2D periapical film images (Figure 8(a)), the images captured using the new method obtained more information from the tooth, and the images were unaffected by compression of the 3D structure of the tissue into a 2D periapical film image (Figure 8(a)), meaning that the images could still show the anteroposterior relationship of the tooth. Mammography also uses tomosynthesis to capture images by scanning at limited angles to obtain reconstructed images [26, 27]; these limited angles mean that the reconstructed slice images are not always clear. The farther from the rotation axis the slice images are taken, the more blurred the slice images become.

In this study, the milliampere seconds per projection was 1.0 mAs for a total exposure of 31 mAs, which is twice of the 15.75 mAs achieved by the previous stationary intraoral digital tomosynthesis system developed by Shan et al. [10]. However, the X-ray tube output for the total exposure in the present study (31 mAs) was half that of another digital tomosynthesis system developed by Ziegler et al. [9], which was 67.2 mAs. Regardless, the X-ray tube output of all intraoral digital tomosynthesis systems should be much less than that of dental CBCT.

The frame rate of a CMOS sensor can be affected by many factors, such as pixel size, X-ray output power, and design of the sensor (e.g., fill factor, quantum efficiency, and signal processing). The intraoral periapical sensor developed in the present study could capture images at 15 Hz, which was sufficient for our 2.5D periapical radiography system. In addition, from the experimental results, the image quality of the intraoral periapical sensor developed in this study was of reasonable quality. However, several aspects should be refined for potential clinical use. The primary concern is that this study used a single tooth to conduct the experiment; thus, no bones or other teeth around the tooth were present to interfere with the images. However, if the system is applied to clinical use, the sensor will be placed in the patient's mouth, which means that there will be many bones and hard tissue of adjacent teeth around the target tooth. This makes the shooting conditions more complex than that of a single tooth. More robust experiments should be performed to verify whether the shooting conditions affect the image quality. In addition, the scanning region of this system is smaller than that of a 2D periapical X-ray due to the X-ray tube requiring exposure in different positions (e.g., a range between ±30°), resulting in the scanning width being less than approximately 80% of the 2D periapical X-ray. Furthermore, the computer operation interface of the high-frame-rate intraoral periapical sensor is relatively complicated; the interface should be improved to increase user friendliness in the future, making system operation easier for dentists and medical image radiologists.

## 5. Conclusion

The developed high-frame-rate intraoral periapical sensor requires further improvement for use in capturing images in a patient's mouth. However, the sensor can greatly reduce shooting time using our 2.5D periapical radiography system to less than 5 s, proving its potential for use in future clinical applications.

## Figures and Tables

**Table 1:** The characteristic features of the high-frame-rate intraoral periapical sensor. Measurements of trabecular bone microarchitectural parameters based on the micro-CT and dental CBCT images.

Number	Item	Specification
1	Process	UMC 0.35 *μ*m CIS with stitching (8 inches)
2	Frame resolution	1008 × 1500
3	Sensitive area	20.16 mm × 30 mm
4	Pixel size	20 *μ*m
5	Output type	Serial
6	Interface (chip to video processing board)	Analog
7	Interface (video processing board to system)	CameraLink
8	Color	Gray
9	Frame rate (max)	≤15 Hz
10	Pixel data rate	15 MHz
11	Pixel sampling resolution	16384 (14 bits)
12	Voltage	3.3 V
13	Power of chip	165 mW
14	Number of pads	68
15	Chip size	20.68 mm × 32.92 mm

## Data Availability

The datasets generated during the current study are available from the corresponding author on reasonable request.
